# A Review of Factors That Influence Individual Compliance with Mass Drug Administration for Elimination of Lymphatic Filariasis

**DOI:** 10.1371/journal.pntd.0002447

**Published:** 2013-11-21

**Authors:** Alison Krentel, Peter U. Fischer, Gary J. Weil

**Affiliations:** 1 Infectious Diseases Division, Department of Internal Medicine, Washington University School of Medicine, St. Louis, Missouri, United States of America; 2 Faculty of Infectious and Tropical Diseases, London School of Hygiene and Tropical Medicine, London, United Kingdom; Fetzer Insittute, United States of America

## Abstract

**Background:**

The success of programs to eliminate lymphatic filariasis (LF) depends in large part on their ability to achieve and sustain high levels of compliance with mass drug administration (MDA). This paper reports results from a comprehensive review of factors that affect compliance with MDA.

**Methodology/Principal Findings:**

Papers published between 2000 and 2012 were considered, and 79 publications were included in the final dataset for analysis after two rounds of selection. While results varied in different settings, some common features were associated with successful programs and with compliance by individuals. Training and motivation of drug distributors is critically important, because these people directly interact with target populations, and their actions can affect MDA compliance decisions by families and individuals. Other important programmatic issues include thorough preparation of personnel, supplies, and logistics for implementation and preparation of the population for MDA. Demographic factors (age, sex, income level, and area of residence) are often associated with compliance by individuals, but compliance decisions are also affected by perceptions of the potential benefits of participation *versus* the risk of adverse events. Trust and information can sometimes offset fear of the unknown. While no single formula can ensure success MDA in all settings, five key ingredients were identified: engender trust, tailor programs to local conditions, take actions to minimize the impact of adverse events, promote the broader benefits of the MDA program, and directly address the issue of systematic non-compliance, which harms communities by prolonging their exposure to LF.

**Conclusions/Significance:**

This review has identified factors that promote coverage and compliance with MDA for LF elimination across countries. This information may be helpful for explaining results that do not meet expectations and for developing remedies for ailing MDA programs. Our review has also identified gaps in understanding and suggested priority areas for further research.

## Author Summary

Lymphatic filariasis (LF, also known as “elephantiasis”) is a deforming and disabling disease that is caused by roundworm parasites that are transmitted by mosquitoes. The Global Programme to Eliminate Lymphatic Filariasis is the largest public health intervention program attempted to date based on mass drug administration (MDA). MDA does not cure filarial infections, but it can reduce or interrupt transmission of new infections by clearing larval parasites from human blood so that they are not available for mosquitoes. High levels of participation are required for this strategy to work; guidelines from the World Health Organization call for at least 65% of the eligible population to take the medications annually for four to six years. MDA presents logistical challenges that require cooperation between donors, health ministries, and communities. The success of MDA depends on coverage (drug delivery) and compliance (people ingesting antifilarial drugs), which depends on individual interactions between drug distributors and the people who live in LF-endemic areas. This paper focuses on this last step of implementation with a comprehensive review of published and unpublished information on factors that affect compliance with MDA at the level of the individual. We have also provided an outline of current gaps in understanding and recommendations for further research.

## Introduction

The Global Programme to Eliminate Lymphatic Filariasis (GPELF) is one of the most ambitious, exciting, and challenging public health programs of our time. Part of the challenge is in the large numbers of people that either have lymphatic filariasis (LF) (an estimated 120 million) or who are at risk for the disease (1.39 billion) [1], [2]. Like many other neglected tropical diseases (NTDs), LF disproportionately affects vulnerable populations and perpetuates existing relationships between disease and poverty [3]. The economic effects of LF can be devastating, as sufferers with disfigurement and disability due to lymphedema, hydrocele, and elephantiasis have reduced work capacity and household income [4]. The ripple effects of this loss of income limit the ability to pay for healthcare, education, and basic household expenses. The social effects of the disease can be equally devastating, potentially ostracizing people from their communities and families [5].

In 1997, the World Health Assembly targeted LF for elimination as a public health problem by the year 2020 [6]. The main tool for GPELF is repeated, annual mass drug administration (MDA) of antifilarial drugs to people living in endemic areas [7], [8]. According to guidelines put forward by the World Health Organization (WHO), at least 65% of the at-risk population should comply with annual MDA so that elimination targets can be reached within four to six years (WHO-GPELF). Implementation of MDA requires cooperation and coordination of activities by donors, national and local health officials, non-governmental organizations (NGOs), and communities.

GPELF is currently active in 53 of 73 countries that are endemic for LF [9]. More than 536 million people received MDA in 2011, and this success depended on the coordinated efforts of the donor community, health ministries, community volunteers, NGOs, and research institutions [9]. GPELF guidelines require high compliance with MDA, but in practice this can be difficult to achieve and sustain. It can be challenging to convince people who feel well to take repeated doses of medicines that may cause adverse events [10]. Emerging challenges to GPELF include program fatigue, knowing what to do when elimination targets have not been reached after six years of MDA, identifying and targeting systematic non-compliers, and maintaining momentum and focus for LF elimination during the new era of integrated NTD control programs.

Securing participation with MDA is essential if GPELF is to achieve the 2020 target for LF elimination. As the program enters its 14^th^ year, the relevance of understanding how to reach individuals and convince them to comply with MDA has become increasingly important. While several countries have stopped MDA and started verification procedures and post-MDA surveillance [9], many LF-endemic countries are still providing MDA or preparing to start. A better understanding of factors that affect compliance with MDA at the level of the individual could have far-reaching effects as programs strive to adapt their campaigns to more effectively reach their target populations.

Many articles have been published that address the issue of compliance with MDA for LF. This paper reports results of a thorough review of publications and unpublished information on this important subject. The goal of this review was to attempt to identify factors and patterns that are associated with compliance with MDA that apply across countries and cultures. We have also attempted to identify high-priority topics for additional research on compliance. Improved understanding of factors that affect an individual's compliance should be helpful not only for LF elimination activities but also for integrated NTD programs that employ MDA and/or preventive chemotherapy.

## Methods

A systematic search of 13 databases including PubMed, Medline, EMBASE, and CAB Abstracts was performed using the key words “compliance,” “non-compliance,” “predictor,” “factor,” “acceptance,” or “refusal” *and* “lymphatic filariasis,” “elephantiasis,” or “filarial” *and* “MDA,” “treatment,” “chemotherapy,” or “treatment coverage.” Papers published in English or French from 2000 through March 2012 were considered. The search generated 404 citations, and a preliminary review of title and abstract was performed using a flowchart for the first screening. In the second review, 86 papers were read in full. Those papers that met one of the following criteria were included in the final dataset: i) reviewed the literature on compliance with MDA for LF; ii) described or assessed factors associated with compliance with MDA for LF; iii) analyzed, observed, or documented compliance rates with MDA and/or provided an explanation or discussion of the rates; and iv) were identified from reference lists of primary papers. In addition to the published literature, informal interviews were conducted with five senior LF scientists who were selected based on their broad international experience and knowledge of issues surrounding compliance. These phone discussions provided the opportunity to solicit unpublished data and reports for inclusion in this review.

A data extraction form was created using Microsoft Excel to enable a systematic analysis of the relevant themes arising from the literature.

### Definitions

For the purposes of this paper, coverage is defined as the percentage of targeted persons who receive MDA medications, and compliance refers to the percentage of a targeted population who swallow the medications. Unless otherwise stated, MDA refers to mass drug administration at the community level; and low or inadequate compliance is described as <65%. See Table 1 for more details of definitions used in this paper.

**Table 1:** Annotated list of definitions for mass drug administration (MDA) programs used by the World Health Organization and the research community [84].

Term	Definition	Source and authors' comments
At-risk population	“Total population in the endemic implementation unit(s).”	[84] This number includes the population eligible and ineligible for MDA.
Directly observed treatment (DOT)	The only method to assure an individual swallowed a drug or a combination of drugs.	[47], [87] If DOT was not performed, only self-reported compliance or surveyed coverage can be reported.
Drug coverage	“Proportion of individuals, expressed as a percentage, in a targeted population who swallowed a drug, or a combination of drugs.”	[84] This paper uses the GPELF definition for drug coverage as the definition for drug compliance.
Epidemiological drug coverage (program coverage)	“Proportion of individuals in the implementation unit who have ingested the MDA drugs of the total population in the implementation unit.”	[76], [84], [88], [89] These papers use the GPELF definition for epidemiological drug coverage as epidemiological drug compliance. This describes the proportion of individuals (%) in an at-risk population who swallowed a drug or a combination of drugs and is used for epidemiological modeling.
Geographical coverage	“Proportion of administrative units that are implementing MDA of all those that require MDA.”	[84] The definition is clear and describes drug distribution independently from drug ingestion.
Ineligible population	“Group of individuals not qualified or entitled to receive anti-helminthic treatment in preventive chemotherapy interventions. Ineligibility is usually determined by exclusion criteria based on drug safety.”	[84] Members of the ineligible population may be infected, and their parasites may result in continued transmission.
National coverage	“Proportion of individuals in an endemic country requiring MDA for LF who have ingested the appropriate drugs.”	[84] Unfortunately, this is a theoretical definition, because programs normally report doses delivered rather than doses ingested. Furthermore, programs with high national coverage may have low epidemiological coverage if ineligible populations are large.
Reported coverage	“Intervention coverage calculated from data reported by all drug distributors.”	[84] This number is often much larger than drug coverage or surveyed coverage.
Surveyed coverage	“A method used to verify reported coverage through use of population-based cluster survey methods. It is calculated as the total number of individuals identified by household survey as having ingested the drugs of the total number of individuals residing in all the surveyed households about whom information on drug ingestion could be elicited.”	[84] Coverage surveys rely on self-reporting by participants. Results may be affected by incomplete recall or by participants' assumptions about answers surveyors want to hear.
Target population for MDA	The population in an implementation unit that is targeted for treatment. This includes those who are eligible to receive the drugs based on safety criteria.	[84] The target population plus the ineligible population form the total at-risk population in an endemic area.

## Results

The search of published literature produced 79 papers that met criteria for inclusion in this review. Unpublished literature and data were provided from studies performed in four different countries (Haiti, Dominican Republic, Cameroon, and Egypt) for review. Additional data on compliance were contributed from small surveys conducted in Malaysia, Togo, Mali, and Niger.

### Study Characteristics

Types of study design included self-reported questionnaires, coverage surveys, household surveys, and intervention studies. Countries where published research was performed included (in order of most to least frequent): India (n = 40), Haiti (n = 7), Sri Lanka (n = 5), Papua New Guinea (n = 5), Kenya (n = 4), Indonesia (n = 3), Tanzania (n = 2), Sierra Leone (n = 1), Vanuatu (n = 1), Ghana (n = 1), Togo (n = 1), American Samoa (n = 1), Egypt (n = 1), and the Philippines (n = 1). Six papers included in the review were from multi-country or non-specific settings.

### Factors Associated with Coverage and Compliance

The reviewed publications used varied definitions for coverage and compliance. Fifteen studies provided quantitative results for factors demonstrated to be associated with compliance (Table S1). However, the majority of the papers reviewed provided qualitative results and anecdotal data for factors associated with compliance (Table S2).

Low MDA compliance may be related to the MDA delivery system and/or to characteristics of targeted recipients. For the purposes of this paper, the delivery system refers to those who distribute the MDA drugs at various levels (national, provincial, district, community, and individual drug distributors), and recipients are those individuals who are targeted to receive MDA in an endemic area. Results are presented according to their relation to the delivery or recipient side of the MDA equation.

### Program and Delivery Issues

Problems with availability of drugs and/or promotional materials were cited as reasons for inadequate coverage in several studies [11]–[14]. For example, inadequate drug supply contributed to low MDA coverage rates in Vanuatu [15] and in East Godavari district in India [16]. Inappropriate distribution time was sometimes cited as negatively affecting coverage. In one Indian study, MDA was postponed and rescheduled to take place during a major Hindu fasting festival, and this made it difficult for distributors to reach individuals for directly observed treatment (DOT) [12]. In another Indian study from Kerala state, repeated postponement of the MDA resulted in poor coverage and high rates of non-compliance [17]. In other cases, insufficient time and personnel adversely affected coverage and compliance [13], [14], [18], [19].

The absence of eligible recipients of the drugs during MDA due to short- or long-term migration provided challenges to coverage in some areas [12], [19], [20]. However, absence at the time of drug distribution was also a commonly reported cause for non-compliance by persons who were not migrants [11], [12], [15], [21]–[31]. It seems that the time allocated for MDA and for mopping-up activities was sometimes not sufficient to reach even those people who were not away at the time of drug distribution [16], [18], [32].

Good coverage in one year sometimes had a positive effect on coverage in subsequent years. One study reported that health workers in the Philippines were especially motivated to reach their coverage target as a result of seeing a reduction of LF cases in the community [33]. In the same study, those individuals who had awareness about LF (p = 0.01) and awareness about MDA (p = 0.02) as well as awareness that MDA was associated with LF (p = 0.01) were more likely to have received the drug. Other factors that were positively associated with good coverage included good coordination among health staff [11] and the introduction of Filaria Prevention Assistants (FPA) or community drug distributors (CDD) to augment the distribution potential of the health services [13], [18], [34], [35].

#### Delivery mechanisms

A number of factors related to details of how drugs were delivered affected compliance in the reviewed studies. These included the identity of the drug distributor, the time of day MDA was carried out, the use of community organizations, the training of the health workers and drug distributors, motivation of health staff, and the use of DOT. These elements were repeatedly cited in reviewed studies as having an impact on compliance with MDA.

Drug distributors are at the interface between MDA programs and their target populations. Characteristics of these frontline workers were identified as a key factor regarding compliance with MDA. Compliance tended to be positively affected when the drug distributor came from the area where he/she was distributing the drugs [16] or when recipients personally knew the drug distributor [26]. Conversely when drug distributors came from a different group or caste, individuals were reluctant to comply [36]. The positive motivation provided by drug distributors was often cited as an important factor that affected compliance with MDA [22], [24], [25]. For example, compliance was positively affected when drug distributors took the medication directly in front of the community [16]. Similarly, if drug distributors visited the household at least one time (or more) prior to MDA, this increased the probability of drug uptake in those households [13].

Some papers reported that direct participation of health personnel in drug distribution positively influenced MDA [36], [37]. In the Philippines, surveyed individuals reported that they complied because they had accepted the health worker's advice [33]. Similarly, in one study from India, compliance was 5.6 times (95% confidence interval (CI) 3.4–9.1) better when MDA was distributed by staff from a local health center [38]. Interactions between health workers and the community sometimes motivated village leaders to participate stressing the “necessity of their cooperation” [16].

The involvement of community organizations and other preexisting networks in MDA distribution sometimes had positive or negative effects on compliance. In several sites, the involvement of community groups or networks in the distribution of drugs improved compliance, because they were able to reach community members efficiently and effectively and thereby enhance participation [11], [39], [40]. The Department of Health in American Samoa evaluated the territory's MDA campaign after inadequate compliance rates (49%) were reported. Following the department's recommendations, subsequent MDAs incorporated churches in the promotion and distribution of the tablets, and this significantly improved compliance rates to between 65% and 86% over the subsequent four years of the program [41]. In contrast to this experience, Aswathy et al. reported that people's fears were exacerbated when members of local self-help groups in India acted as drug distributors, because they were too hurried to answer people's concerns about adverse events [42]. Low motivation by drug distributors was also mentioned as contributing to low compliance in a report from another area in India, where overburdened local health workers considered the MDA program to be an unwelcome additional duty [16]. Similarly in a study from Kenya, drug distributors cited lack of supervision from health staff, short training, lack of incentives, and delays in supplies as reasons for low motivation [43].

Inadequate training of health workers and drug distributors can have negative effects on MDA compliance. Some reports mentioned that pills were sometimes dropped off at the house [11] or left for family members to distribute without an adequate explanation [12], [14]. In a study from Sri Lanka, individuals reported that they did not like receiving drugs from volunteers, because the volunteers lacked information about the drugs. Also, in some cases volunteers were considered to be too young to be distributing drugs. In the same study, volunteer distributors might have had more credibility if they had arrived with a badge or an official letter [26]. In an Indian study, drug distributors' poor communication skills were credited with contributing to poor compliance as people doubted their ability to assess eligibility for DEC [38]. While some of these issues may not have been directly related to poor training, improved training and supervision may have improved performance.

The private health sector can have an important influence on compliance with MDA. One study from India reported that private practitioners exaggerated the possible adverse events of MDA, and this discouraged people from taking the pills [44]. Other reports mentioned that when medical practitioners were not involved in the MDA process, they remained unaware of the benefits and necessity of MDA and sometimes advised their patients against compliance [45], [46]. In other cases, community participation was positively affected when practitioners took the pills in public and actively supported the program (authors' unpublished observations).

Although directly observed drug administration is recommended for MDA programs (DO-MDA), it was infrequently used in the studies reviewed for this paper. MDA programs tended to be highly successful when DO-MDA was systematically employed [34], [41], [47]. One of the difficulties of ensuring DO-MDA was the perceived challenge of reaching individuals after meals so they could avoid taking the medications on an empty stomach [13], [14], [37].

#### MDA medication issues

Many studies reported concerns from individuals about the pills provided during MDA as reasons for non-compliance. The large number of pills to be swallowed was cited most frequently, and this was followed by complaints about the size and taste of the pills [13], [15], [16], [48]–[50]. There were also concerns about the quality of the drugs. One report from Haiti mentioned that the pills were “bad” [23], while one paper from India mentioned that the pills had disintegrated into powder [30]. The distribution of “loose tablets” was also cited as a reason not to comply with MDA [17], [22].

### Individual Recipient Characteristics

#### Awareness and knowledge

One of the most prominent factors associated with motivating compliance and increasing coverage was advance knowledge of the MDA, as demonstrated in studies from India, Sri Lanka, the Philippines, Sierra Leone, and Vanuatu [15], [18], [25], [26], [33], [51], [52]. The inverse of this was also observed, when individuals did not comply because they were unaware of the MDA or the LF elimination program [13], [19], [22], [53].

Across the literature, knowledge associated with compliance was primarily related to three specific topics: transmission of LF, knowledge that MDA protects against LF, and knowledge of lymphedema management techniques. Knowing that mosquitoes are responsible for the transmission of LF was shown to be associated with compliance in several studies [24], [26]–[28], [51], [54]. Ramaiah et al. described the inverse of this phenomena whereby people were unwilling to comply because they reported that they controlled mosquitoes in their own living environments and therefore were not at risk for LF [55]. Those individuals who reported knowing that MDA medications prevented LF were also more likely to comply [13], [15], [16], [22], [24]–[26], [33], [39], [42], [53].

In studies that performed multivariate analysis, knowledge that MDA was for LF was a key factor associated with compliance [24], [25]. One Indian study reported a strong association between compliance and the interaction between knowing about MDA in advance and knowing that everyone was at risk for LF (odds ratio (OR) 16.1; 95% CI 8.8–29.3) [25]. Another Indian study demonstrated that the combination of advanced knowledge of MDA and knowing that mosquitoes transmit LF dramatically increased the probability of a person complying with MDA [24].

Finally, there was evidence that knowledge of lymphedema management techniques and the availability of case management programs for diseased individuals (lymphedema management programs and hydrocele surgery) improved compliance with MDA [25], [35], [51]. For example, establishment of and support for Hope Clubs in Cap Haitien, Haiti helped to demonstrate that the LF elimination program had a long-term commitment to help those affected by LF and that it was not simply a short-term drug administration program (L. R. Carpenter, personal communication).

Misconceptions and misinformation about the treatment inhibited people's uptake of MDA medications. For example, some people in the Philippines believed that the treatment might cause sterility, fainting, or even death [33]. Uptake of MDA increased after these misconceptions were corrected. In Tanzania, misperceptions about MDA resulted in lower compliance rates; specifically, some people believed the drugs were a contraceptive while others thought that the pills inhibited libido [56]. Similar fears that the drugs were contraceptives were expressed in Kenya [34]. In Vanuatu, some believed that the pills would cause LF and that the government was trying to sterilize the communities [15]. Compliance also suffered in areas where people were misinformed about the etiology of LF (believing it to be due to heredity or caused by a curse) [13] (A. Viall, personal communication, and authors' unpublished observations).

Aside from the three primary knowledge indicators discussed above, there was scant information on further associations between knowledge and compliance. Detailed knowledge regarding LF and MDA was not always associated with high compliance with MDA in some areas (Els Mathieu, personal communication, and authors' unpublished observations [57], [58]).

#### Understanding the personal risk of LF and the benefits of MDA

hose individuals who were able to express their own personal risk for LF were more likely to comply with MDA. In some papers, this was described as having seen someone with LF or having someone in the family with LF, while in other cases it was expressed as an understanding that “everyone is at risk for LF” [25], [26], [51]. One study from Sri Lanka demonstrated higher compliance in urban areas for those who had seen someone with clinically evident LF [26]. The inverse of this perception was also demonstrated. That is to say, those who did not perceive LF to be a problem in their community were less likely to comply with MDA [12], [59], [60].

Those who were able to personalize the benefit of the MDA for their own individual health were likely to be compliers [16], [22], [39], [42]. Perceived benefit was positively associated with compliance in studies from India and the Philippines [33], [42]. Understanding that the MDA was beneficial was similarly associated with increased compliance [26]. Note that perceived benefit of the treatment is not the same as understanding that the MDA is for LF.

#### Adverse events and MDA

The potential for adverse events following MDA was responsible for discouraging as well as encouraging uptake. Many papers cited individuals in their surveys who were afraid of adverse events from the treatment and as a result did not want to comply with MDA [11], [13]–[18], [22]–[26], [30], [31], [33], [37], [50], [59], [61]–[63]. Some of this fear arose from inadequate explanations about possible adverse events and a failure to understand that severe adverse events are rare [42].

Rumors regarding adverse events associated with MDA can seriously affect compliance by individuals. Three studies from India and one from Haiti mentioned erroneous reports of deaths due to MDA that negatively affected compliance [11], [28], [39], [44]. When these rumors were not addressed, they persisted and negatively affected MDA in neighboring areas as well [12], [64].

When communities did not receive correct and adequate information about adverse events, the consequences were detrimental to the success of the MDA. In Orissa, India, insufficient information about the MDA and its possible adverse events led to an erosion of confidence in the community, and as a result, the MDA was postponed for three years [65]. In East Godavari district, India, MDA was suspended due to adverse events until health workers intervened and addressed community concerns [16]. On the other hand, in areas where the management of adverse events was monitored and well coordinated in communities, individuals cited this as positively influencing their acceptance of MDA and suggested that the possibility of adverse events did not inhibit their participation in MDA [16], [66]. Similarly there were some instances where those who experienced adverse events following MDA in one year were more likely to comply the following year [67], [68]. Anecdotal evidence from Alor district, Indonesia suggested that some individuals equated adverse events with evidence that the treatment was effective [57]. However, adverse events following MDA in one year sometimes deterred individuals from compliance in subsequent years [27], [67].

The elimination of intestinal helminths was cited in several papers as a positive consequence of MDA [69], [70]. This deworming effect was perceived to be a community benefit, and this contributed to overall community acceptance of the MDA program. For example, Talbot et al. found that Haitian subjects who did not know that the pills contained albendazole (ALB) were five time more likely to be non-compliant than those who did know about ALB, suggesting that the people were aware of the collateral deworming benefits of this medication [71]. Anecdotal reports from Indonesia, Papua New Guinea, and Haiti described individuals who were grateful or even “excited” when they or their children passed intestinal worms following MDA (L. R. Carpenter, personal communication, and authors' unpublished observations).

#### Recipient's personal situation

Several publications mentioned that personal characteristics influenced compliance with MDA. Characteristics cited included the individual's personal health at the time of drug distribution, whether or not they were taking other medications, their gender, age, household income, or residence in urban or rural areas, and as mentioned above, their understanding of their own personal risk for infection with LF. Individual health status and current use of medication heavily affected people's compliance with MDA. In many instances, papers cited “healthy,” “have no LF disease,” or “drugs are unnecessary” as reasons for non-compliance among their study populations [11], [13], [14], [16]–[19], [22], [25], [26], [30], [37], [39], [53], [61]–[63]. Being ill at the time of MDA was also cited as a reason for non-compliance [11], [16], [22], [24], [25], [27], [37], [39], [42]. Furthermore, it was also commonly expressed that people refused MDA because they were presently taking other medications (including contraceptives) and that they were concerned about possible interactions with the LF drugs [18], [23], [26], [28], [30], [37], [61]. Treatment for other illnesses was the primary reason given for systematic non-compliance in one study [39].

Female gender was often associated with lower rates of compliance with MDA. For example, pregnancy was often cited as a reason for non-compliance, as pregnant women were not eligible for MDA, and lactation was considered to be a contraindication to MDA in the early years of the program. In Haiti, women of childbearing age were excluded at the beginning of the MDA campaign due to fears of using ALB in early pregnancy. Although this policy was reversed prior to the third round of MDA, it had a lasting effect, because women continued to have low compliance rates [71]. Other reasons cited for low MDA compliance in Haitian women included a rumor that women should not comply with MDA if they were taking contraception, fear of miscarriage, and a desire to avoid embarrassment at MDA distribution posts where they would be asked if they were pregnant or not (N. Barkey, unpublished report). In some household situations, women also were prohibited from seeking or taking MDA, because of their husbands' negative beliefs regarding the treatment (authors' unpublished observations).

Age was closely associated with MDA compliance in some studies. In most cases, the youngest (under 5 years of age) were the group most frequently cited as non-compliers, because their parents feared how the treatment might affect their children [14], [23], [30], [72]. In one study from Egypt, children aged 2–4 years showed lower rates of compliance than adults. However, this may have been due to blood tests conducted at the time of the survey [73]. In a few studies, the elderly were also reported to be systematic non-compliers [39], [40] as were individuals 15–34 years of age in a Ghanaian study [40] and people aged 16–30 years in an unpublished report from Haiti (N. Barkey [2009]). Similarly, Wynd et al. discovered that the younger generation in Papua New Guinea was becoming resistant to continuing compliance in some areas, because they thought that LF was no longer a threat [74].

Area of residence and household income often affected compliance. Living in a rural environment was associated with higher compliance in several studies [11], [13], [37], [75], while achieving compliance in urban areas was more challenging. Inadequate coverage and compliance in urban areas were attributed to some of the following factors: lack of a specific urban strategy [11], [72], fewer peripheral health workers [72] and volunteers [26], the dominance of private health care providers [22], [55], poor health care infrastructure [22], [55], and the presence of unorganized settlements and large numbers of migrants [55]. Individuals with lower incomes were also more receptive to MDA in some urban areas [55], while those with higher incomes were more difficult to reach and to convince to take the medications [39], [48].

## Discussion

A better understanding of factors that influence compliance with MDA for LF may improve results for current and future MDA campaigns. This paper has attempted to provide a thorough review of published information on this topic in the global context, and that information was supplemented by unpublished data and interviews with key informants.

It is important to emphasize the importance of definitions and the distinction between coverage (delivery of the medicines) and compliance (ingestion of the pills). For example, the WHO collects reported coverage data from national LF elimination programs. However, GPELF publications sometimes use reported coverage data as if they were the same as “coverage with ingestion of drugs” [9]. Another illustration of the importance of these definitions comes from the computer simulation literature. The LYMFASIM simulation model uses “the fraction of people treated per round” (equivalent to “epidemiological drug coverage” as defined in Table 1) as a key input for estimating the impact of MDA on filarial infection rates and transmission [76]. Important differences between “epidemiological drug coverage” and “reported coverage” may explain the persistence of LF after several years of MDA in countries despite high reported coverage rates.

One of the limitations of this review is that its primary focus is on the factors influencing compliance decisions at the individual level. Little published information is available on how factors such as program design, management, or implementation might affect coverage and compliance. Hence, further research is needed in this area.

The reviewed studies were quite varied in terms of size, location, and design. Results also varied, and our challenge was to tease out the common themes. Our review did not identify a specific compliance recipe that will work in any country or context. However, the study has identified five key “ingredients” that appear to be essential for encouraging compliance at the individual level for successful MDA programs.

### 1. Acknowledging the Role of Trust in MDA

**Figure 1:**
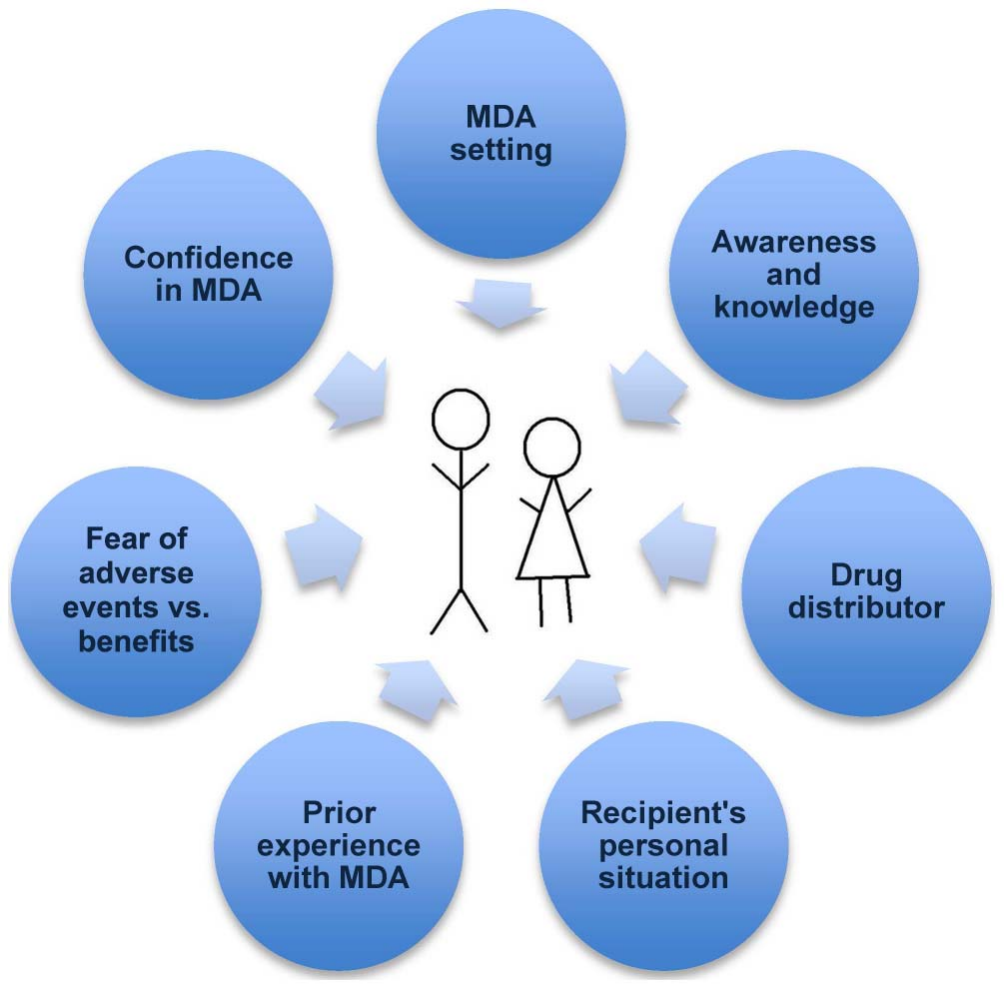
Major factors that affect individual compliance with mass drug administration for elimination of lymphatic filariasis.

The issue of trust permeates many of the factors and specific experiences that are associated with compliance or non-compliance. Specifically, individuals need to trust the person(s) delivering the drugs to them and communities need to trust the health workers and governments promoting and directing the MDA. As a result, elimination programs need to ensure that elements of trust are built into campaigns so as to effectively engage with communities. Some of these elements are outlined in Figure 1 and discussed below.

It is impossible to overemphasize the importance of the face-to-face interaction between the drug distributor and the individual who receives the medicine. Recipients may be reassured, motivated, discouraged, angered, or confused during this critical interaction. We found that the identity of the drug distributor was consistently linked to compliance. The following factors regarding distributors may build trust in MDA: selection of distributors known to the community and with reputations or other credentials to indicate that they can be trusted, adequate training for communicating knowledge related to MDA, motivation and belief in the importance of the program, and adequate time for MDA and a willingness to answer questions. While it is recognized that the role of the drug distributor is key to the efforts to eliminate LF, more work is needed to develop protocols to motivate and sustain the commitment of drug distributors over the long course of MDA programs.

Government health workers serve as drug distributors in some communities, and as stated above, many of the same issues apply to their success at the point of distribution. Since community members trust their advice, they tend to be successful promoters of MDA if they provide motivation, have the time to reassure participants about adverse events, and if they are enthusiastic about the campaign. Compliance suffers when they are overburdened, unmotivated, and undertrained. Private health providers could provide a similar service, however they are usually not systematically involved in the promotion and administration of the MDA. Engagement of private providers could reduce misinformation or confusing messages from these workers about MDA programs and improve community compliance. Published literature and WHO guidelines do not describe proven strategies to enhance motivation and participation by public or private health care workers in MDA programs.

At the onset of MDA programs, consideration of several programmatic components is warranted in order to cultivate trust between the health services and the recipient communities—namely timely promotion of MDA, adequate management of adverse events, and recognition of other pressing health needs in the community. Each of these elements has been shown to enhance individual compliance with MDA. Across international contexts, advance knowledge of MDA was positively associated with ingestion of the pills. In order to promote MDA effectively and to encourage the establishment of the social norm of compliance, information about the pills and their distribution needs to be made frequently available before MDA begins to foster an environment of trust between the delivery system and the recipient. For communities to be effectively engaged in the elimination of LF, evidence shows that they need to hear about the MDA not only from the health services, but also from local leaders (cultural, religious, village), household authority figures, and from others in their personal networks. The involvement of these persons in MDA enhances confidence in the MDA program and further promotes its individual and collective benefits.

In 2000 when the GPELF was launched, it stood on two pillars—MDA and morbidity management. In the majority of the papers reviewed here, there was little discussion or evidence that morbidity management had been implemented as a complement to MDA. From evidence in India, Haiti, Indonesia, and Zanzibar, it is clear that lymphedema management programs and hydrocele surgeries lend credibility to MDA programs. Aside from the benefit to MDA, there is clear evidence that these programs also alleviate the suffering caused by the long-term manifestations of LF and tangibly improve the lives of patients and their families [77].

Many articles mentioned that fear motivates many people not to take the tablets. If the components discussed above can be put into place before MDA begins, then this can help to alleviate fears and anxieties by establishing an environment of trust to increase chances for a successful program.

### 2. Tailoring a Distribution System That Is Appropriate to the Local Environment

MDA occurs in many different contexts around the world and also within countries and regions. These contexts reflect varied histories, local culture, and the impact of local leaders. Programs that strive to work together with leaders and that try to understand and respect their beliefs, trust networks, and fears will have better chances for success. In addition, programs should be planned so that MDA begins only after drugs, promotional materials, and personnel are available and in place. Delays in distribution can confuse communities and provide opportunities for rumors to arise and persist that may hamper compliance with MDA when it finally arrives. Social scientists have recommended comprehensive anthropological studies as prerequisites to MDA [78]. While the results of these studies may well enhance the planning of MDA, time, money, and expertise are not always available to carry them out. When such studies are not feasible, programs can look to other health agencies working in the same area to learn from their experiences (immunization drives, polio campaigns, maternal and child health programs) and adapt the MDA accordingly.

While several studies have emphasized the importance of DO-MDA, it is more the exception than the rule within GPELF. More effort in this area is needed to ensure that individuals actually swallow the tablets that are distributed to reduce the gap between coverage and compliance. Planners should consider the habits of community members, their work, and social schedules including meal times. Drug distributors must be trained on procedures to follow when they encounter non-compliers. Options might include referral to a supervisor or scheduling a time for a second visit when there is more time for questions and discussion. This type of activity increases the time and effort for drug distribution, and this should be taken into account when planners consider time and budgets for drug distribution.

### 3. Management of Adverse Events

Adverse events (AEs) following MDA are generally mild, and the frequency of AEs decreases after the first round of MDA [79]. However, our literature review showed that fear of AEs is one of the major reasons people do not comply with MDA. Furthermore, residual apprehension about the possibility of AEs may persist in an area even after the frequency of such events has declined [23]. As a result, programs should be vigilant when planning MDA and in the early years of MDA to ensure that health services are prepared to handle minor AEs as they arise. Management of AEs in these first years of treatment will benefit the program in subsequent years, as this will reassure communities that the drugs are safe and that the health services can be trusted. In the same vein, community drug distributors need be aware of how AEs will be managed so they can clearly communicate plans for this with communities and individuals as they distribute the pills.

The positive side effects (so-called “ancillary benefits”) of MDA need to be communicated and reiterated during the pre-MDA socialization as well as at the time of pill distribution. The clearance of soil-transmitted helminths is a major associated benefit of MDA, particularly in children. Drug distributors should convey this information to community leaders and to families so that they can use this information to increase acceptance of MDA.

### 4. Promotion of Other “Non-Health” Benefits of Compliance

Traditionally, awareness campaigns stress the functional benefits of a cure or treatment that heals sickness or prevents disease. Generally speaking, health officials and workers consider health to be an adequate motivation to achieve participation or compliance. However, the promise of health may not be a strong motivator in areas where clinically evident LF is uncommon. In many of the papers reviewed, the prospect of being cured or preventing LF was infrequently named as the primary reason people complied with treatment.

While the health benefits of MDA may indeed influence some to take the treatment, the non-health benefits may be equally influential on behavior. Therefore campaigns should make every effort to promote intangible benefits associated with the treatment. These might include: feeling modern by accepting non-traditional medicines, being perceived as someone who cares about his family and health, being seen as someone who fits in with others in the community, being a good citizen and following the government program, being smart and preventing future economic loss, protecting future generations from LF, or feeling safer. Promotion of additional benefits of MDA may stimulate demand for the tablets.

To illustrate this further, the association between LF and a cycle of poverty has been well documented [80]. Those living with chronic filariasis suffer from acute attacks that require them to forgo work for a period of time and seek medical assistance. Those with elephantiasis, lymphedema, or hydrocele may also suffer from associated stigma [5], [81]; the emotional burden of these conditions is only just starting to be understood [82]. With this knowledge in mind, promotion of MDA should address the economic and social benefits of treatment. Understanding the everyday economic benefits of LF elimination at the household level may persuade some individuals to comply. Furthermore, when discussing LF elimination with government health officials, the importance of these economic savings over the long term for endemic districts should be part of the promotion and advocacy activities.

Children under five years of age were identified as systematic non-compliers in several studies from areas where MDA is provided to children starting as young as two years of age. Publicity regarding the deworming effects of MDA (with the associated improvements in general health, school attendance, and growth) may be useful for countering this problem. If parents understand that MDA has these benefits in addition to anti-LF properties, this may tip the balance in favor of participation. It may also be helpful to explain that the time-limited MDA program aims to provide a healthier and safer environment for all future children and generations. For example, a paper published in 2011 stated that MDA had already protected 66 million newborns from acquiring LF [83].

### 5. Addressing the Issue of Systematic Non-Compliance

Systematic non-compliers have been identified as persons who persistently refuse or do not ingest antifilarial medications over the multi-year course of an MDA program. Non-compliers can serve as a continued source of infection that may place their community at continued risk for transmission of LF. This has been demonstrated in Haiti and in Egypt, where those who reported never taking MDA had higher infection rates than compliant persons [23], [47]. Therefore further work is needed to identify systematic non-compliers and their motivations. Explanations will vary in different locations, but special attention should be paid to the issues of seasonal migration and low compliance in young children. When groups with high rates of systematic non-compliance are identified, specific approaches are needed to counter factors that result in non-compliance. There is an urgent need to develop guidelines for managing this problem, as none currently exist.

### Areas Recommended for Further Research

#### Concepts of coverage and compliance

Since definitions and equations for coverage and compliance varied in the papers reviewed for this study, it was challenging to compare results and experiences across different papers. It would be helpful if researchers and national programs could agree to a uniform set of definitions. The definitions in the most recent guidelines from WHO [84] (reprinted in Table 1) are a good starting point. However, we believe that the definitions in this document should be modified to clarify the distinction between coverage and compliance. Most of the publications reviewed for this paper use the term “compliance” to describe what is called “drug coverage” in the guidelines. As this contributes to confusion, the authors propose that the term “compliance” should be included as another term for “drug coverage” in the guidelines to improve clarity and understanding.

#### A “tool box” for difficult areas

LF elimination is progressing well in many countries. However, special approaches and innovative methods may be required for countries or areas that are not meeting expectations. These often face special challenges such as difficult access, post-conflict health systems and populations, post-natural-disaster environments, large urban settings, and fragmented societies. They may include areas that have failed transmission assessment surveys (TAS) despite many years of MDA. New tools are needed to help LF programs to quickly assess the characteristics of these challenging environments and plan MDA or other responses accordingly. When standard approaches are not achieving the goal, other resources (private sector or other donors) and implementation partners (faith-based organizations, NGOs) may tip the balance toward success. By approaching these more challenging areas intentionally and differently, a troubleshooting guide may be helpful to guide assessment, execution of MDA, monitoring, and evaluation. A basic troubleshooting guide is presented in Table 2 with suggested tools. This can be adapted for local use, and it should be modified over time based on feedback regarding its use.

**Table 2:** A basic troubleshooting guide for commonly encountered problems in MDA programs.

Scenario	Description of issue	Suggested areas for intervention
Low reported coverage	*Drugs are not reaching targeted population.*	1. Who are drug distributors—are they appropriate for the community?
		2. Check motivation of drug distributors in terms of incentives, training, logistical capacity.
		3. Assess security situation and time of MDA.
		4. Is the distribution method appropriate?
		5. Check reporting forms and systems.
High reported coverage with low drug coverage (e.g., compliance with treatment)	*Drugs distributed to targeted population are not being swallowed.*	1. Consider how DO-MDA can be implemented (time of day for MDA, distribution method).
		2. Assess level of awareness about MDA in the community; should novel communications be introduced (cell phones, social media)?
		3. Are local leaders and groups involved in the process?
		4. Assess fear and management of adverse events.
		5. Evaluate role and reputation of health services in the population.
High rates of systematic non-compliance	*Individuals have not ingested the drugs in any MDA round.*	1. Identify subgroups with high non-compliance rates and design programs to target these groups.
		2. Consider a test-and-treat approach.
		3. Use of behavior change models to address persistent non-compliance (e.g., motivational interviewing).
Local health system (at IU level) weak and unable to conduct MDA	*Local health system weakened due to lack of personnel, funds, or post-conflict situation.*	1. Identify local NGOs or organizations who would be capable of conducting MDA in a specific IU.
		2. Where logistics are challenged, identify possible private sector participants to fill the gap.
Reported coverage decreasing as MDA rounds continue	*Drugs are not reaching the population as MDA continues.*	1. Consider health service personnel and drug distributor fatigue and how to address it.
		2. Has there been a change in local management?
		3. Has there been a change in logistical provision?
Drug coverage decreasing as MDA rounds continue	*Individuals who may have taken drugs in previous rounds are refusing to comply with treatment.*	1. Has there been a population increase to affect the denominator?
		2. Are there persistent rumors affecting the campaign?
		3. Are adverse events being adequately managed?
		4. Assess drug distributors' ability to respond to questions, fears.
High reported coverage and low surveyed coverage	*Drug distributors report distributing adequate number of drugs to the targeted population, but independent coverage surveys report lower rates of drug coverage or compliance.*	1. Check reporting forms and systems.
		2. Review possible population shifts.
		3. Consider how DO-MDA can be implemented (time of day for MDA, method of distribution).
		4. Assess security situation and timing of MDA.
		5. Is the distribution method appropriate?

#### Gaps in understanding

Areas that have been identified that require additional research fall into four categories: service delivery, strategic issues for NTD programs, and compliance at the level of the community and the individual (Table 3). This review has identified gaps in understanding related to service delivery and program strategy. Some of these are related to the recent move to integrate programs for diverse NTDs that vary with regard to intervention tools, target populations, and goals (control *versus* elimination). More work is needed to improve understanding of the impact of this integration on LF elimination efforts so that the benefits of integration can be harvested while minimizing the risks that integration may pose to GPELF.

**Table 3:** Research needs and gaps in understanding.

Key area and topic	Associated questions	Suggested methods
***A. Service delivery***		
1. Relationship between the delivery system and the recipient and how that affects compliance	What is the role of the delivery mechanism in achieving compliance? How to train, supervise, motivate, and empower drug distributors?	Participant observation of MDA, semi-structured interviews, coverage surveys, pilot study
2. Interactions at the point of delivery/distribution	What conditions are necessary to enhance compliance? What happens at the point of distribution?	Participant observation, semi-structured interviews
3. Operational considerations to ensure the use of DO-MDA	What factors must be considered to achieve DO-MDA? Are there best practices to promote?	Literature review, surveys with health staff and community members, pilot study
4. Best practices for MDA	What is working in different contexts? How can this information be collated and shared with program managers to enhance their control efforts?	Identify best practices globally through literature review, discussions with key informants, prepare case studies, disseminate results at scientific meetings
5. Innovative approaches to enhance social mobilization and drug delivery	Explore use of social media, mobile phone technology, NGO networking, outsourcing, use existing networks (e.g., HIV/AIDS infrastructure, community health workers)	Case studies to test new methodologies in a way that can be evaluated and replicated
***B. Strategic issues for LF and other NTD programs***		
1. Integration of the lessons learned from GPELF into NTD programs	What are the key lessons from 12 years of GPELF? How might they be applied to NTD programs?	Literature review, interviews with key informants
2. Sharing of research results and operational knowledge to and in between members of the GPELF community	How is knowledge shared between programs? How can sharing be enhanced?	Key informant interviews, exploration of new formats to share information
3. Understanding the changing dynamics of six or more years of MDA in a community/district/country	What changes might programs expect over time (fatigue, misperceptions, funding, societal changes) and how to address those?	Document experiences from MDA programs after six or more years and how they adapted to changing conditions
4. Focus on difficult and challenging environments for MDA and identify solutions for improved coverage and compliance	What are the current difficult environments? What tools can be used to reach these people? How can populations be segmented for mobilization?	Pilot studies
5. Integration of LF elimination and NTD programs	How should social mobilization reflect NTD integration? M&E? How does introduction of other NTD programs affect ongoing MDA for LF?	Literature review, key informant interviews
***C. Compliance and the individual***		
1. The impact of systematic non-compliers (SNC) and how to reach them	Who are SNC? What are their characteristics? What can be done to convince them to comply with MDA?	Literature review, key informant interviews, pilot studies to test specific interventions
***D. Compliance and the community***		
1. Understanding the impact of morbidity management on compliance	Why does morbidity management influence compliance? How can it be promoted and sustained? Does it have the same effect across contexts?	Literature review, key informant interviews
2. Engaging the community in an urban environment	What social groups can be activated for MDA in urban areas? What is the best way to conduct DO-MDA in these environments?	Pilot studies, literature review

## Final Conclusions

There is a sense of urgency to some of the issues raised in this paper. The clock is ticking for the elimination of LF by 2020. Through unprecedented pharmaceutical donations, the drugs for MDA are ready and available for use by national programs. The focus should now move from the issue of supply to the question of how to best deliver precious donated drugs into the mouths of those living in endemic areas. The success of the global program hinges on sufficient and sustained compliance, which is achieved one person at a time. The risks of insufficient compliance are too great to ignore. These include the possible emergence of drug resistance [85], the potential need for additional rounds of treatment [76] with their associated costs, and the risk of program fatigue at the community and health service levels. With seven years remaining, focused attention is needed to optimize MDA in countries that are just starting their programs and to improve ongoing campaigns in countries where compliance has been less than adequate. Finally, in light of the London Declaration of 2012 [86] and the increased global commitment to eliminate or control NTDs, it will be important to share and integrate lessons learned from various NTD programs regarding compliance and behavioral change to maximize the benefit of these interventions for at-risk populations.

## Supporting Information

Table S1 **Summary of statistically supported results from key papers demonstrating factors associated with compliance.**
(DOCX)

Table S2 **Summary of qualitative results from key papers demonstrating factors associated with compliance.**
(DOCX)

## References

[1] WHO (2011) Global programme to eliminate lymphatic filariasis: progress report on mass drug administration, 2010. Wkly Epidemiol Rec 86: 377–388. 21887884

[2] Hotez PJ, Molyneux DH, Fenwick A, Kumaresan J, Ehrlich Sachs S, et al (2007) Control of neglected tropical diseases. New Engl J Med 357: 1018–1027. 17804846 10.1056/NEJMra064142

[3] Hotez PJ, Fenwick A, Savioli L, Molyneux D (2009) Rescuing the bottom billion through control of neglected diseases. Lancet 373: 1570–1575. 19410718 10.1016/S0140-6736(09)60233-6

[4] Zeldenryk LM, Gray M, Speare R, Gordon S, Melrose W (2011) The emerging story of disability associated with lymphatic filariasis: a critical review. PLoS Negl Trop Dis 5: e1366 doi:10.1371/journal.pntd.0001366 22216361 10.1371/journal.pntd.0001366 PMC3246437

[5] Perera M, Whitehead M, Molyneux D, Weerasooriya M, Gunatilleke G (2007) Neglected patients with a neglected disease? A qualitative study of lymphatic filariasis. PLoS Negl Trop Dis 1: e128 doi:10.1371/journal.pntd.0000128 18060080 10.1371/journal.pntd.0000128 PMC2100378

[6] (1997) Elimination of lymphatic filariasis as a public health problem. Document WHA50/1997/REC/1.

[7] Ottesen EA, Duke BO, Karam M, Behbehani K (1997) Strategies and tools for the control/elimination of lymphatic filariasis. Bull World Health Organ 75: 491–503. 9509621 PMC2487030

[8] Ottesen EA (2000) The global programme to eliminate lymphatic filarisis. Trop Med Int Health 5: 591–594. 11044272 10.1046/j.1365-3156.2000.00620.x

[9] WHO (2012) Global programme to eliminate lymphatic filariasis: progress report, 2011. Wkly Epidemiol Rec 87: 346–356. 22977953

[10] Partono F, Maizels RM, Purnomo (1989) Towards a filariasis-free community: evaluation of filariasis control over an eleven year period in Flores, Indonesia. Trans R Soc Trop Med Hyg 83: 821–826. 2617653 10.1016/0035-9203(89)90343-x

[11] Babu B, Kar SK (2004) Coverage, compliance and some operational issues of mass drug administration during the programme to eliminate lymphatic filariasis in Orissa, India. Trop Med Int Health 9: 702–709. 15189460 10.1111/j.1365-3156.2004.01247.x

[12] Lahariya C, Mishra A (2008) Strengthening of mass drug administration implementation is required to eliminate lymphatic filariasis from India: an evaluation study. J Vector Borne Dis 45: 313–320. 19248659

[13] Ramaiah KD, Vijay Kumar KN, Hosein E, Krishnamoorthy P, Augustin DJ, et al (2006) A campaign of ‘communication for behavioural impact’ to improve mass drug administrations against lymphatic filariasis: structure, implementation and impact on people's knowledge and treatment coverage. Ann Trop Med Parasitol 100: 345–361. 16762115 10.1179/136485906X105598

[14] Ranganath BG (2010) Coverage survey for assessing mass drug administration against lymphatic filariasis in Gulbarga district, Karnataka, India. J Vector Borne Dis 47: 61–64. 20231778

[15] Fraser M, Taleo G, Taleo F, Yaviong J, Amos M, et al (2005) Evaluation of the program to eliminate lymphatic filariasis in Vanuatu following two years of mass drug administration implementation: results and methodologic approach. Am J Trop Med Hyg 73: 753–758. 16222021

[16] Babu BV, Satyanarayana K (2003) Factors responsible for coverage and compliance in mass drug administration during the programme to eliminate lymphatic filariasis in the East Godavari District, South India. Trop Doct 33: 79–82. 12680538 10.1177/004947550303300208

[17] Showkath Ali MK, Rajendran R, Regu K, Mohanan MK, Dhariwal AC, et al (2007) Study on the factors affecting the MDA programme in Kerala state. J Commun Dis 39: 51–56. 18338717

[18] Weerasooriya MV, Yahathugoda CT, Wickramasinghe D, Gunawardena KN, Dharmadasa RA, et al (2007) Social mobilisation, drug coverage and compliance and adverse reactions in a Mass Drug Administration (MDA) Programme for the Elimination of Lymphatic Filariasis in Sri Lanka. Filaria J 6: 11. 18005398 10.1186/1475-2883-6-11 PMC2203982

[19] Ramaiah KD, Das PK, Appavoo NC, Ramu K, Augustin DJ, et al (2000) A programme to eliminate lymphatic filariasis in Tamil Nadu state, India: compliance with annual single-dose DEC mass treatment and some related operational aspects. Trop Med Int Health 5: 842–847. 11169272 10.1046/j.1365-3156.2000.00659.x

[20] Sunish IP, Rajendran R, Mani TR, Gajanana A, Reuben R, et al (2003) Long-term population migration: an important aspect to be considered during mass drug administration for elimination of lymphatic filariasis. Trop Med Int Health 8: 316–321. 12667150 10.1046/j.1365-3156.2003.01033.x

[21] Anthony G, Deming M, Dorkenoo AM, Morgah K, Verani J, et al (2009) The integration of neglected diseases: three years of experience in Togo. Am J Trop Med Hyg 81: 327–328.

[22] Babu BV, Mishra S (2008) Mass drug administration under the programme to eliminate lymphatic filariasis in Orissa, India: a mixed-methods study to identify factors associated with compliance and non-compliance. Trans Roy Soc Trop Med Hyg 102: 1207–1213. 18632125 10.1016/j.trstmh.2008.05.023

[23] Boyd A, Won KY, McClintock SK, Donovan CV, Laney SJ, et al (2010) A community-based study of factors associated with continuing transmission of lymphatic filariasis in Leogane, Haiti. PLoS Negl Trop Dis 4: e640 doi:10.1371/journal.pntd.0000640 20351776 10.1371/journal.pntd.0000640 PMC2843627

[24] Cantey PT, Rao G, Rout J, Fox LM (2010) Predictors of compliance with a mass drug administration programme for lymphatic filariasis in Orissa State, India 2008. Trop Med Int Health 15: 224–231. 20002615 10.1111/j.1365-3156.2009.02443.x

[25] Cantey PT, Rout J, Rao G, Williamson J, Fox LM (2010) Increasing compliance with mass drug administration programs for lymphatic filariasis in India through education and lymphedema management programs. PLoS Negl Trop Dis 4: e728 doi:10.1371/journal.pntd.0000728 20628595 10.1371/journal.pntd.0000728 PMC2900179

[26] Gunawardena S, Ismail M, Bradley M, Karunaweera N (2007) Factors influencing drug compliance in the mass drug administration programme against filariasis in the Western province of Sri Lanka. Trans R Soc Trop Med Hyg 101: 445–453. 17125809 10.1016/j.trstmh.2006.09.002

[27] Mathieu E, Direny AN, Beau de Rochars M, Streit TG, Addiss DG, et al (2006) Participation in three consecutive mass drug administrations in Leogane, Haiti. Trop Med Int Health 11: 862–868. 16772008 10.1111/j.1365-3156.2006.01626.x

[28] Mathieu E, Lammie PJ, Radday J, Beach MJ, Streit T, et al (2004) Factors associated with participation in a campaign of mass treatment against lymphatic filariasis, in Leogane, Haiti. Ann Trop Med Parasitol 98: 703–714. 15509424 10.1179/000349804X3135

[29] Mitjà O, Paru R, Hays R, Griffin L, Laban N, et al (2011) The impact of a filariasis control program on Lihir Island, Papua New Guinea. PLoS Negl Trop Dis 5: e1286 doi:10.1371/journal.pntd.0001286 21886851 10.1371/journal.pntd.0001286 PMC3160343

[30] Regu K, Showkath Ali MK, Rajendran R, Koya SM, Ganesh B, et al (2006) Mass drug administration against lymphatic filariasis: experiences from Kozhikode district of Kerala State. J Commun Dis 38: 333–338. 17913209

[31] Ray Karmakar P, Mitra K, Chatterjee A, Jana PK, Bhattacharya S, et al (2011) A study on coverage, compliance and awareness about mass drug administration for elimination of lymphatic filariasis in a district of West Bengal, India. J Vector Borne Dis 48: 101–104. 21715733

[32] Das PK, Pani SP, Krishnamoorthy K (2002) Prospects of elimination of lymphatic filariasis in India. ICMR Bulletin 32: 14.

[33] Amarillo ML, Belizario VY Jr, Sadiang-Abay JT, Sison SA, Dayag AM (2008) Factors associated with the acceptance of mass drug administration for the elimination of lymphatic filariasis in Agusan del Sur, Philippines. Parasit Vectors 1: 14. 18505577 10.1186/1756-3305-1-14 PMC2441609

[34] Wamae N, Njenga SM, Kisingu WM, Muthigani PW, Kiiru K (2006) Community-directed treatment of lymphatic filariasis in Kenya and its role in the national programmes for elimination of lymphatic filariasis. Afr J Health Sci 13: 69–79. 17348745 10.4314/ajhs.v13i1.30819

[35] Mohammed KA, Molyneux DH, Albonico M, Rio F (2006) Progress towards eliminating lymphatic filariasis in Zanzibar: a model programme. Trends Parasitol 22: 340–344. 16713740 10.1016/j.pt.2006.05.010

[36] Ramaiah KD, Vijay Kumar KN, Chandrakala AV, Augustin DJ, Appavoo NC, et al (2001) Effectiveness of community and health services-organized drug delivery strategies for elimination of lymphatic filariasis in rural areas of Tamil Nadu, India. Trop Med Int Health 6: 1062–1069. 11737843 10.1046/j.1365-3156.2001.00813.x

[37] Nujum ZT (2011) Coverage and compliance to mass drug administration for lymphatic filariasis elimination in a district of Kerala, India. International Health (RSTMH) 3: 22–26. 10.1016/j.inhe.2010.12.001 24038047

[38] Mahalakshmy T, Kalaiselvan G, Parmar J, Dongre A (2010) Coverage and compliance to diethylcarbamazine in relation to Filaria Prevention Assistants in rural Puducherry, India. J Vector Borne Dis 47: 113–115. 20539050

[39] Nandha B, Sadanandane C, Jambulingam P, Das P (2007) Delivery strategy of mass annual single dose DEC administration to eliminate lymphatic filariasis in the urban areas of Pondicherry, South India: 5 years of experience. Filaria J 6: 7. 17718908 10.1186/1475-2883-6-7 PMC2020462

[40] Gyapong M, Gyapong JO, Owusu-Banahene G (2001) Community-directed treatment: the way forward to eliminating lymphatic filariasis as a public-health problem in Ghana. Ann Trop Med Parasitol 95: 77–86. 11235557 10.1080/00034980020035942

[41] King JD, Zielinski-Gutierrez E, Pa'au M, Lammie P (2011) Improving community participation to eliminate lymphatic filariasis in American Samoa. Acta Trop 120S: S48–S54. 10.1016/j.actatropica.2010.08.021 20932818

[42] Aswathy S, Beteena K, Leelamoni K (2009) Mass drug administration against filariasis in India: perceptions and practices in a rural community in Kerala. Ann Trop Med Parasitol 103: 617–624. 19825283 10.1179/000349809X12459740922255

[43] Njomo DW, Amuyunzu-Nyamongo M, Magambo JK, Ngure PK, Njenga SM (2012) Factors associated with the motivation of community drug distributors in the Lymphatic Filariasis Elimination Programme in Kenya. South Afr J Epidemiol Infect 27: 66–70.

[44] Babu BV (2010) A qualitative study on the adverse reactions of mass treatment for lymphatic filariasis in Orissa, India. Asian Pacific J Trop Med 3: 55–58.

[45] Babu BV, Nath N (2004) The programme to eliminate lymphatic filariasis in Orissa, India: the attitudes of some programme partners. Ann Trop Med Parasitol 98: 751–757. 15509429 10.1179/000349804225021433

[46] Kerketta AS, Babu BV (2009) Clinician's attitude on mass drug administration under the program to eliminate lymphatic filariasis: a qualitative study from Orissa, India. Asia Pac J Public Health 21: 112–117. 19124342 10.1177/1010539508327032

[47] El-Setouhy M, Elaziz KMA, Helmy H, Farid HA, Kamal HA, et al (2007) The effect of compliance on the impact of mass drug administration for elimination of lymphatic filariasis in Egypt. Am J Trop Med Hyg 77: 1069–1073. 18165524 PMC2196399

[48] Njomo D, Nyamongo M, Njenfa S, Magambo J (2010) Socio-economic and behavioural factors that influence compliance with mass treatment in the national programme for elimination of lymphatic filariasis in Kenya. Am J Trop Med Hyg 83: 67–67.

[49] Rajendran R, Sunish IP, Mani TR, Munirathinam A, Abdullah SM, et al (2002) The influence of the mass administration of diethylcarbamazine, alone or with albendazole, on the prevalence of filarial antigenaemia. Ann Trop Med Parasitol 96: 595–602. 12396322 10.1179/000349802125001726

[50] Sapak P, Melrose W, Durrheim D, Pawa F, Wynd S, et al. (2004) Evaluation of the lymphatic filariasis control program Samarai Murua District, Papua New Guinea. Warwick, Queensland: Warwick Educational Publishing.

[51] Cantey PT, Rao G, Rout J, Jolly A, Williamson J, et al (2009) Increased adherence to mass drug administration for lymphatic filariasis - Orissa State, India, 2009. Am J Trop Med Hyg 91: 293–294.

[52] Hodges MH, Smith SJ, Fussum D, Koroma JB, Conteh A, et al (2012) High coverage of mass drug administration for lymphatic filariasis in rural and non-rural settings in the Western Area, Sierra Leone. Parasit Vectors 3: 120. 10.1186/1756-3305-3-120 PMC3018440 21162751

[53] Vaishnav KG, Patel IC (2006) Independent assessment of Mass Drug Administration in filariasis affected Surat city. J Commun Dis 38: 149–154. 17370677

[54] Njomo DW, Amuyunzu-Nyamongo M, Mukoko DA, Magambo JK, Njenga SM (2012) Socioeconomic factors associated with compliance with mass drug administration for lymphatic filariasis elimination in Kenya: descriptive study results. Ann Trop Med PH 5: 103–110.

[55] Ramaiah KD, Vijay Kumar KN, Ravi R, Das PK (2005) Situation analysis in a large urban area of India, prior to launching a programme of mass drug administrations to eliminate lymphatic filariasis. Ann Trop Med Parasitol 99: 243–252. 15829134 10.1179/136485905X29701

[56] Mackenzie CD, Lazarus WM, Mwakitalu ME, Mwingira U, Malecela MN (2009) Lymphatic filariasis: patients and the global elimination programme. Ann Trop Med Parasitol 103: S41–S51. 19843397 10.1179/000349809X12502035776630

[57] Krentel A, Fischer P, Manoempil P, Supali T, Servais G, et al (2006) Using knowledge, attitudes and practice (KAP) surveys on lymphatic filariasis to prepare a health promotion campaign for mass drug administration in Alor District, Indonesia. Trop Med Int Health 11: 1731–1740. 17054754 10.1111/j.1365-3156.2006.01720.x

[58] Njomo DW, Amuyunzu-Nyamongo M, Mukoko DA, Magambo JK, Njenga SM (2012) Social mobilization and compliance with mass treatment for lymphatic filariasis elimination in Kenya. Afr J Health Sci 20: 42–49.

[59] Kumar A, Kumar P, Nagaraj K, Nayak D, Ashok L, et al (2009) A study on coverage and compliance of mass drug administration programme for elimination of filariasis in Udupi district, Karnataka, India. J Vector Borne Dis 46: 237–240. 19724089

[60] Pattanshetty S, Kumar A, Kumar R, Rao CR, Badiger S, et al (2010) Mass drug administration to eliminate lymphatic filariasis in Southern India. AMJ 3: 847–850.

[61] Kasturiratne KT, Premaratne BA, Pathmeswaran A, de Silva NR, de Silva HJ (2001) Compliance with the mass chemotherapy program for lymphatic filariasis. Ceylon Med J 46: 126–129. 12164029 10.4038/cmj.v46i4.6431

[62] Krentel A, Aunger R (2011) Causal chain mapping: a novel method to analyse treatment compliance decisions relating to lymphatic filariasis elimination in Alor, Indonesia. Health Policy Plan 384–395. 21712348 10.1093/heapol/czr048

[63] Showkath AM, Regu K, Rajendran R, Mohanan MK, Ganesh B (2008) Awareness of health personnel about lymphatic filariasis and mass drug administration in Kerala State. J Commun Diseases 40: 37–40. 19127667

[64] Ramaiah KD, Ravi R, Das PK (2005) Preventing confusion about side effects in a campaign to eliminate lymphatic filariasis. Trends Parasitol 21: 307–308. 15923141 10.1016/j.pt.2005.05.015

[65] Babu BV, Satyanarayana K (2003) Healthcare workers' knowledge of lymphatic filariasis and its control in an endemic area of Eastern India: implications on control programme. Trop Doct 33: 41–42. 12568522 10.1177/004947550303300120

[66] Bhullar N, Maikere J (2010) Challenges in mass drug administration for treating lymphatic filariasis in Papua, Indonesia. Parasit Vectors 3: 70. 20701744 10.1186/1756-3305-3-70 PMC2928210

[67] Bockarie MJ, Tisch DJ, Kastens W, Alexander ND, Dimber Z, et al (2002) Mass treatment to eliminate filariasis in Papua New Guinea. New Engl J Med 347: 1841–1848. 12466508 10.1056/NEJMoa021309

[68] Vanamail P, Ramaiah KD, Subramanian S, Pani SP, Yuvaraj J, et al (2005) Pattern of community compliance with spaced, single-dose, mass administrations of diethylcarbamazine or ivermectin, for the elimination of lymphatic filariasis from rural areas of southern India. Ann Trop Med Parasitol 99: 237–242. 15829133 10.1179/136485905X29666

[69] de Rochars MB, Direny AN, Roberts JM, Addiss DG, Radday J, et al (2004) Community-wide reduction in prevalence and intensity of intestinal helminths as a collateral benefit of lymphatic filariasis elimination programs. Am J Trop Med Hyg 71: 466–470. 15516644

[70] Mani TR, Rajendran R, Munirathinam A, Sunish IP, Md Abdullah S, et al (2002) Efficacy of co-administration of albendazole and diethylcarbamazine against geohelminthiases: a study from South India. Trop Med Int Health 7: 541–548. 12031078 10.1046/j.1365-3156.2002.00894.x

[71] Talbot JT, Viall A, Direny A, de Rochars MB, Addiss D, et al (2008) Predictors of compliance in mass drug administration for the treatment and prevention of lymphatic filariasis in Leogane, Haiti. Am J Trop Med Hyg 78: 283–288. 18256430

[72] Babu BV, Behera DK, Kerketta AS, Mishra S, Rath K, et al (2006) Use of an inclusive-partnership strategy in urban areas of Orissa, India, to increase compliance in a mass drug administration for the control of lymphatic filariasis. Ann Trop Med Parasitol 100: 621–630. 16989688 10.1179/136485906X118521

[73] Weil GJ, Kastens W, Susapu M, Laney SJ, Williams SA, et al (2008) The impact of repeated rounds of mass drug administration with diethylcarbamazine plus albendazole on bancroftian filariasis in Papua New Guinea. PLoS Negl Trop Dis 2: e344 doi:10.1371/journal.pntd.0000344 19065257 10.1371/journal.pntd.0000344 PMC2586652

[74] Wynd S, Durrheim DN, Carron J, Selve B, Chaine JP, et al (2007) Socio-cultural insights and lymphatic filariasis control - lessons from the Pacific. Filaria J 6: 1–4. 17306032 10.1186/1475-2883-6-3 PMC1805494

[75] Gunawardena GS, Ismail MM, Bradley MH, Karunaweera ND (2007) Impact of the 2004 mass drug administration for the control of lymphatic filariasis, in urban and rural areas of the Western province of Sri Lanka. Ann Trop Med Parasitol 101: 335–341. 17524248 10.1179/136485907X176364

[76] Stolk WA, de Vlas SJ, Borsboom GJ, Habbema JD (2008) LYMFASIM, a simulation model for predicting the impact of lymphatic filariasis control: quantification for African villages. Parasitology 135: 1583–1598. 19006602 10.1017/S0031182008000437

[77] Mand S, Debrah AY, Klarmann U, Batsa L, Marfo-Debrekyei Y, et al (2012) Doxycycline improves filarial lymphedema independent of active filarial infection: a randomized controlled trial. Clin Infect Dis 55: 621–630. 22610930 10.1093/cid/cis486 PMC3412691

[78] Wynd S, Melrose WD, Durrheim DN, Carron J, Gyapong M (2007) Understanding the community impact of lymphatic filariasis: a review of the sociocultural literature. Bull World Health Organ 85: 493–498. 17639248 10.2471/BLT.06.031047 PMC2636343

[79] Hochberg N, Michel MC, Lammie PJ, Mathieu E, Direny AN, et al (2006) Symptoms reported after mass drug administration for lymphatic filariasis in Leogane, Haiti. Am J Trop Med Hyg 75: 928–932. 17123989

[80] Hotez PJ, Fenwick A, Savioli L, Molyneux DH (2009) Rescuing the bottom billion through control of neglected tropical diseases. Lancet 373: 1570–1575. 19410718 10.1016/S0140-6736(09)60233-6

[81] Wijesinghe RS, Wickremasinghe AR, Ekanayake S, Perera MS (2007) Physical disability and psychosocial impact due to chronic filarial lymphoedema in Sri Lanka. Filaria J 6: 4. 17391538 10.1186/1475-2883-6-4 PMC1851956

[82] Litt E, Baker MC, Molyneux D (2012) Neglected tropical diseases and mental health: a perspective on comorbidity. Trend Parasitol 28: 195–201. 10.1016/j.pt.2012.03.001 22475459

[83] Molyneux DH, Malecela MN (2011) Neglected tropical diseases and the millennium development goals-why the “other diseases” matter: reality versus rhetoric. Parasit Vectors 4: 234. 22166580 10.1186/1756-3305-4-234 PMC3271994

[84] WHO (2011) Monitoring and epidemiological assessment of mass drug administration in the global programme to eliminate lymphatic filariasis: a manual for national elimination programmes. Geneva, Switzerland.

[85] Smits HL (2009) Prospects for the control of neglected tropical diseases by mass drug administration. Expert Rev Anti Infect Ther 7: 37–56. 19622056 10.1586/14787210.7.1.37

[86] www.unitedtocombatntds.org (2013) From promises to progress: the first annual report on the London Declaration on NTDs. Available: http://www.unitingtocombatntds.org/reports/promises-to-progress-EN.pdf. Accessed 30 October 2013.

[87] Kyelem D, Biswas G, Bockarie MJ, Bradley MH, El-Setouhy M, et al (2008) Determinants of success in national programs to eliminate lymphatic filariasis: a perspective identifying essential elements and research needs. Am J Trop Med Hyg 79: 480–484. 18840733 PMC2694403

[88] Michael E, Malecela-Lazaro MN, Maegga BT, Fischer P, Kazura JW (2006) Mathematical models and lymphatic filariasis control: monitoring and evaluating interventions. Trends Parasitol 22: 529–535. 16971182 10.1016/j.pt.2006.08.011

[89] Stolk WA, ten Bosch QA, de Vlas SJ, Fischer PU, Weil GJ, et al (2013) Modeling the impact and costs of semiannual mass drug administration for accelerated elimination of lymphatic filariasis. PLoS Negl Trop Dis 7: e1984 doi:10.1371/journal.pntd.0001984 23301115 10.1371/journal.pntd.0001984 PMC3536806

