# Supplementary material for: A Review of Factors That Influence Individual Compliance with Mass Drug Administration for Elimination of Lymphatic Filariasis
Source: PLoS Negl Trop Dis. 2013 Nov 21;7(11):e2447. doi: 10.1371/journal.pntd.0002447 (PMC3836848; doi:10.1371/journal.pntd.0002447)
Supplement: Table S1 — Summary of statistically supported results from key papers demonstrating factors associated with compliance. (DOCX) [file pntd.0002447.s001.docx]

**Table S1. Summary of statistically supported results from key papers demonstrating factors associated with compliance.**

| **Location, year** | **Sample size** | **Factor demonstrated to be associated with compliance** | **P value** | **OR, CI** |
| --- | --- | --- | --- | --- |
| *Univariate analysis* | | | | |
| Haiti, 2008 [[71](#_ENREF_71)] | 367 | Perceived risk of contracting LF | ≤ 0.05 |  |
| India, 2009 [[42](#_ENREF_42)] | 599 | Perceived benefit of MDA to individual  Perceived usefulness of MDA  Certain ward of residence | <0.001  0.001  0.03 |  |
| India, 2011 [[37](#_ENREF_37)] | 1185 | Who the drug administrator was | 0.002 |  |
| India, 2006 [[30](#_ENREF_30)] | 320 HH | Female gender | <0.01 |  |
| Kenya, 2012 [[58](#_ENREF_58)] | 965 | Knowledge of LF  Source of information  Content of information  Frequency of receiving information about MDA | >0.05  <0.001  <0.001  <0.001 |  |
| Kenya, 2012 [[54](#_ENREF_54)] | 965 | Religion  Ownership of land  Knowledge of signs of lymphedema and hydrocele  Perception of risk of LF infection | <0.001  <0.001  <0.001  <0.001 |  |
| Philippines, 2008 [[33](#_ENREF_33)] | 437 | Knowledge of LF  Perceived benefit of antifilarial drugs | 0.08  0.06 |  |
| Sri Lanka, 2007 [[18](#_ENREF_18)] | 4358 | Awareness of MDA | <0.0001 |  |
| Vanuatu, 2005 [[15](#_ENREF_15)] | 1632 | Hearing about MDA in advance  Knowing antifilarial drugs prevent LF | <0.001  <0.001 |  |
| *Multivariate analysis* | | | | |
| Haiti, 2004 [[28](#_ENREF_28)] | 304 | Male gender  Knowing that mosquitoes transmit LF  Having learned about MDA from posters or banners |  | 3.3 (1.5-7.4)  2.6 (1.2-5.4)  2.9 (1.2-7.5) |
| Haiti, 2008 [[71](#_ENREF_71)] | 367 | *Factors associated with noncompliance*  Female gender  Would it be good if people followed my example of noncompliance? (Answer: no)  Do you think the program uses good drugs? (Answer: no/don’t know)  Heard about LF program? (Answer: no)  Can you swallow pill? (Answer: no)  Did not know that pill contained Albendazole | 0.028  <0.001  0.035  0.028  0.002  0.001 | 2.74 (1.12-6.70)  5.74 (2.33-14.14)  3.77 (1.10-12.96)  3.17 (1.14-8.87)  8.56 (2.26-32.46)  5.08 (2.01-12.84) |
| India, 2010 [[38](#_ENREF_38)] | 1282 | Drug distribution from health staff rather than volunteers |  | 5.6 (3.4-9.1) |
| India, 2010 [[24](#_ENREF_24)] | 547 | Knew MDA was for LF  Knew everyone at risk for LF  Knew about MDA in advance  Knew that mosquitoes transmit LF  Interaction of knowing about MDA in advance & that mosquitoes transmit LF |  | 2.6 (1.4-5.1)  1.6 (0.98-2.7)  1.9 (1.1-3.2)  0.8 (0.3-2.1)  5.4 (2.8-10.4) |
| India, 2010 [[25](#_ENREF_25)] | 1269 | Age > 35 years & ≤ 45 years  No school  Grades 1-5  Knew that MDA was for LF  Knew about lymphedema management  Knew about MDA in advance  Knew everyone at risk for LF  Interaction between knew about MDA in advance and knew everyone at risk for LF |  | 2.0 (1.2-3.2)  3.5 (1.2-10.0)  3.2 (1.4-7.3)  3.3 (1.7-6.6)  3.3 (1.6-6.9)  4.8 (3.8-8.1)  2.2 (1.0-4.8)  16.1 (8.8-29.3) |
| Sri Lanka, 2007 [[26](#_ENREF_26)] | 2319 | Rural population rather than urban  Males more compliant than females  Believing MDA was beneficial versus not |  | 0.72 (0.55-0.97)  0.57 (0.43-0.76)  0.26 (0.15-0.47) |
| Sri Lanka, 2001 [[61](#_ENREF_61)] | 1935 | Passed grade 8  Passed General Certificate of Education (Ordinary Level) |  | 1.9 (1.2-2.8)  1.6 (1.1-2.4) |
