# Supplementary material for: A Review of Factors That Influence Individual Compliance with Mass Drug Administration for Elimination of Lymphatic Filariasis
Source: PLoS Negl Trop Dis. 2013 Nov 21;7(11):e2447. doi: 10.1371/journal.pntd.0002447 (PMC3836848; doi:10.1371/journal.pntd.0002447)
Supplement: Table S2 — Summary of qualitative results from key papers demonstrating factors associated with compliance. (DOCX) [file pntd.0002447.s002.docx]

**Table S2. Summary of qualitative results from key papers demonstrating factors associated with compliance.**

| **Study, year, reference** | **Sample size** | **Key findings of factors demonstrated to be associated with compliance (+/-)** |
| --- | --- | --- |
| American Samoa, 2011 [[41](#_ENREF_41)] | 153, 261 in 2 KAP; FGD; 43 KII | Connection with community established through churches for drug distribution (+) |
| Egypt, 2007 [[47](#_ENREF_47)] | 1012, 1025, 1009, 1115 1064 (5 surveys) | Strength of government in social mobilization (+); directly observed MDA (+) |
| Ghana, 2001 [[40](#_ENREF_40)] | 810 | Low compliance amongst 15-34 year olds and elderly (>64 years); lack of health facility reduced compliance in areas where HW responsible for MDA |
| Haiti, 2004 [[28](#_ENREF_28)] | 304 | Know LF a mosquito borne disease (+); male gender (+); encouraging others to comply (+); received message through poster or banner (+); rumors of adverse events (-) |
| Haiti, 2006 [[27](#_ENREF_27)] | 1767; 392; 2177 (3 surveys) | Male gender (+); primary school-aged children (+); know LF transmitted by mosquitoes and knowing tablets prevent disease (+); believed the drug made you ill (-); busy, pregnant, away during MDA (-); unaware of need to comply (-) |
| Haiti, 2008 [[71](#_ENREF_71)] | 367 | Ability to swallow the pills (+); knowledge of LF and MDA (+); no personal benefit perceived from MDA (-); not knowing about Albendazole (-); male gender (+); mistrust in drugs (-) |
| Haiti, 2010 [[23](#_ENREF_23)] | 455 | Fear of adverse events (-); don’t like to take pills (-); low compliance amongst 3-5 year old children (-) |
| India, 2000 [[19](#_ENREF_19)] | 6482; 5 FGD; 19 KII | MDA unnecessary (-); poor publicity (-); poor awareness of benefits of drug (-); aide effects (-); too many tablets (-); lacked confidence in DD (-) |
| India, 2001 [[36](#_ENREF_36)] | 3869 | PHC in village (+); confidence in HW as distributors (+); lack of faith in community leadership (-); group and caste (-); other more pressing basic needs in the community (-) |
| India, 2003 [[16](#_ENREF_16)] | 800 HH; 27 FGD; 25 KII; 45 SSI | Motivation, training and supervision of HW (+); village leaders understood necessity of their cooperation; (+); community-chosen DD (+); MDA after meals (+); too many tablets (-); shortage of drugs (-); lack of motivation of HW (-) |
| India, 2004 [[11](#_ENREF_11)] | 13826; 20 FGD; 113 KII | Living in rural area (+); living in urban area (-) good HW coordination (+); involvement of community groups (+); MDA unnecessary (-); media exaggerated adverse events (-) |
| India, 2005 [[68](#_ENREF_68)] | 13990 | Those developing adverse events in 1^st^ round of treatment more likely to complete all 6 rounds of MDA (+) |
| India, 2006 [[72](#_ENREF_72)] | 7226 in 3 sites | Specific efforts to reach marginalized groups (+); shared management of resources with community (+); 2-4 years less compliant (-) |
| India, 2006 [[53](#_ENREF_53)] | 3640 | Believe MDA is effective to prevent LF (+); fear of LF (+); persuaded by DD (+); empty stomach at time of MDA (-); too many tablets (-); need consent from family doctor or head of family (-); lack of faith in DD (-); rumors (-); adverse events (-) |
| India, 2006 [[30](#_ENREF_30)] | 320 HH | Female gender (+); MDA not necessary; fear of side effects (-); undergoing treatment for other diseases (-); no LF present (-); parents afraid to treat children (-); too many tablets (-); do not like DD (-) |
| India, 2006 [[13](#_ENREF_13)] | 4182 pre and 3862 post intervention; 75 FGD | Prevents LF (+); fear of elephantiasis (+); living in rural areas (+); one or more pre-MDA visits by DD to the HH (+); large number of tablets (-); fear of adverse events (-); unnecessary (-); local beliefs about LF (-); lack of confidence in DD (-) |
| India, 2007[[17](#_ENREF_17)] | 166 | Poor coordination (-); lack of training (-); lack of publicity about MDA (-); postponement of MDA (-); poor quality of the drugs (-); poor remuneration of DD (-); fear of side effects (-); no disease = no need for MDA (-) |
| India, 2007 [[39](#_ENREF_39)] | 1179, 3195, 2976, 3127, 1953 (5 surveys) | Trust in DD (+); afraid to get LF (+); aware of beneficial effect of MDA (+); unnecessary (-); fear of adverse events (-); being elderly (-); undergoing treatment for another illness (-); high income (-) |
| India, 2008 [[12](#_ENREF_12)] | 2173 | Inappropriate IEC media (-); mistrust between HW and community (-); adverse events not well managed (-); negative rumors (-) |
| India, 2008 [[22](#_ENREF_22)] | 2688; 240 SSI | DD and HW convince to consume (+); from government (+); LF in the family (+); free drugs (+/-); fear of adverse events (-) |
| India, 2009 [[59](#_ENREF_59)] | 1145 | Fear of adverse events (-); did not receive drugs (-); LF not considered a problem (-) |
| India, 2009 [[42](#_ENREF_42)] | 599 | Living in certain wards (+); fear (-) |
| India, 2010 [[60](#_ENREF_60)] | 1090 | LF not a serious problem (-); presence of other medical disorders (-) |
| India, 2010 [[14](#_ENREF_14)] | 602 | Did not receive drugs (-); no information (-); MDA unnecessary (-); parents feared giving MDA to children (-); DD did not insist on DOT (-); fear of adverse events (-) |
| India, 2010 [[44](#_ENREF_44)] | 35 FGD and 209 KII | Fear of adverse events (-); private practitioners advised against MDA (-) |
| India, 2010 [[24](#_ENREF_24)] | 3449 HH and 547 in 2 areas | Fear of adverse events (-); MDA unnecessary (-); know MDA prevents LF (+); DD told them to comply (+); know everyone is at risk for LF (+); know mosquitoes transmit LF (+); know MDA in advance (+) |
| India, 2010 [[25](#_ENREF_25)] | 1285 HH; 1269 KAP | Know MDA prevents LF (+); DD or family member told them to comply (+); know MDA in advance (+); knowing one component of lymphedema management (+); fear of adverse events (-); lack of trust in DEC (-) |
| India, 2011 [[37](#_ENREF_37)] | 1185 | Knowing who the DD is (+); fear of adverse events (-); unnecessary (-); taking pills for another condition (-); unacceptability of drug distributor (-) |
| India, 2011 [[31](#_ENREF_31)] | 166 | Living in rural area (+); fear of adverse events ; inadequate counseling about the drug (-); no faith in drug (-) |
| India, 2012 [[38](#_ENREF_38)] | 1282 | Distribution by health centre rather than volunteer (+); motivation of volunteers (+); volunteer’s communication skills (-) |
| Indonesia, 2011 [[62](#_ENREF_62)] | 21 | Social norm of compliance (+); authority (+/-); effect of MDA on household economics (+/-) |
| Kenya, 2006 [[34](#_ENREF_34)] | 720 and 3465 (2 surveys); 882 SSI; 14 FGD | DOT was enforced (+); religious affiliation (-); false rumors about drug’s use (-) |
| Kenya, 2010 [[48](#_ENREF_48)] | 965 HH | Higher income (-); dislike of distribution methods (-); drug itself (taste, size, number of pills) (-) |
| Kenya, 2012 [[54](#_ENREF_54)] | 965; 160 IDI; 16 FGD | Risk perception (+); knowledge of the cause of a swollen limb (+); access to MDA information (+) |
| Papua New Guinea, 2004 [[50](#_ENREF_50)] | 4 villages | Presence of adverse events (-); too many tablets (-); other conditions share same local name as LF and are not affected by MDA (-) |
| Philippines, 2008 [[33](#_ENREF_33)] | 437 | Believed HW advice (+); fear of adverse events (-); inability to work (-) |
| Sierra Leone, 2012 [[52](#_ENREF_52)] | 9249 | Coordinated, intense and focused SM using traditional and modern outlets (+) |
| Sri Lanka, 2001 [[61](#_ENREF_61)] | 1935 | High/very low education levels (-); awareness of asymptomatic LF carriers (+); fear of interaction with other medications (-) |
| Sri Lanka, 2007 [[26](#_ENREF_26)] | 2319 | Not receiving drugs (-); know DD (+); being very poor or very rich (-); male gender (+); living in a rural area (+); believing that MDA is beneficial (+); having a neighbor with LF (+) |
| Sri Lanka, 2007 [[18](#_ENREF_18)] | 4358 | Take MDA after meals at night (+); awareness of MDA (+); under other medication (-); unnecessary (-); adverse events (-); forgot (-); lack of confidence in DD (-); DD lacked motivation (-) |
| Tanzania, 2009 [[56](#_ENREF_56)] | 71 | Improvements to LF patient conditions affects compliance (+); young males believe fertility has increased because of MDA (+); misconceptions about drug (-) |
| Vanuatu, 2005 [[15](#_ENREF_15)] | 1632 KAP; 106 HW | Protect from LF (+); to stop LF spreading in their community (+); hearing in advance about MDA (+); MDA unnecessary (-); false rumors (-); large number of pills (-); fear of adverse events (-); away during MDA (-) |

DD=drug distributors; DOT=directly observed treatment; FGD=focus group discussion; HW=health workers; HH=households; IDI=in depth interviews; IEC=information, education and communication; KAP=Knowledge, Attitudes, Behavior survey; PHC=primary health care; SM= social mobilization; SSI=semi-structured interviews
